# Nitrated nucleosome levels and neuropsychiatric events in systemic lupus erythematosus; a multi-center retrospective case-control study

**DOI:** 10.1186/s13075-017-1495-6

**Published:** 2017-12-22

**Authors:** Isabel Ferreira, Sara Croca, Maria Gabriella Raimondo, Manjit Matharu, Sarah Miller, Ian Giles, David Isenberg, Yiannis Ioannou, John G. Hanly, Murray B. Urowitz, Nicole Anderson, Cynthia Aranow, Anca Askanase, Sang-Cheol Bae, Sasha Bernatsky, Ian N. Bruce, Jill Buyon, Ann E. Clarke, Mary Anne Dooley, Paul Fortin, Ellen Ginzler, Dafna Gladman, Caroline Gordon, Murat Inanc, Søren Jacobsen, Kenneth Kalunian, Diane Kamen, Munther Khamashta, Sam Lim, Susan Manzi, Joan Merrill, Ola Nived, Christine Peschken, Michelle Petri, Rosalind Ramsey-Goldman, Guillermo Ruiz-Irastorza, Jorge Sanchez-Guerrero, Kristjan Steinson, Gunnar K. Sturfelt, Ronald van Vollenhoven, Daniel J. Wallace, Asad Zoma, Anisur Rahman

**Affiliations:** 1 Centre for Rheumatology Research, University College London, Fourth Floor Rayne Institute, 5 University Street, London, WC1E 6JF UK; 2 Headache Group, Institute of Neurology and The National Hospital for Neurology and Neurosurgery, Queen Square, London, UK; 3 Arthritis Research UK Centre for Adolescent Rheumatology, UCL/UCLH/Great Ormond Street Hospital, London, UK; 4 Division of Rheumatology, Dalhousie University and Queen Elizabeth II Health Sciences Center, Halifax, NS Canada; 5 Lupus Program, Centre for Prognosis Studies in The Rheumatic Disease and Krembil Research Institute, Toronto Western Hospital, University of Toronto, Toronto, ON Canada; 6 Feinstein Institute for Medical Research, Manhasset, NY USA; 7 Rheumatology, Columbia University, New York, NY USA; 8 Department of Rheumatology, Hanyang University Hospital for Rheumatic Diseases, Seoul, South Korea; 9 Divisions of Clinical Epidemiology and Rheumatology, McGill University Health Centre, Montreal, QC Canada; 10 Arthritis Research UK Centre for Epidemiology, Centre for Musculoskeletal Research, The University of Manchester, and NIHR Manchester Musculoskeletal Biomedical Research Unit, Central Manchester University Hospitals NHS Foundation Trust, Manchester Academic Health Science Centre, Manchester, UK; 11 New York School of Medicine, New York, NY USA; 12 Division of Rheumatology, Cumming School of Medicine, University of Calgary, Calgary, AB Canada; 13 Thurston Arthritis Research Center, University of North Carolina, Chapel Hill, NC USA; 14 Centre Hospitalier de l’Université Laval (CHUL), Québec, QC Canada; 15 Downstate Medical Center Rheumatology, Brooklyn, New York, NY USA; 16 Rheumatology Research Group, Institute of Inflammation and Ageing, College of Medical and Dental Sciences, University of Birmingham, Birmingham, UK; 17 Department of Internal Medicine, Istanbul University, Istanbul, Turkey; 18 Copenhagen Lupus and Vasculitis Clinic, Centre For Rheumatology and Spine Diseases, Rigshospitalet, Copenhagen, Denmark; 19 University of California, San Diego, La Jolla, CA USA; 20 Division of Rheumatology and Immunology, Medical University of South Carolina, Charleston, SC USA; 21 FRCP Division of Women’s Health, King’s College, London, UK; 22 Department of Medicine, Emory University, Atlanta, GA USA; 23 Allegheny Health Network, Pittsburgh, PA USA; 24 Clinical Pharmacology, Oklahoma Medical Research Foundation, Oklahoma City, OK USA; 25 Department of Rheumatology, Lund University, Lund, Sweden; 26 Department of Medicine, Rady Faculty of Health Sciences, University of Manitoba, Winnipeg, MB Canada; 27 Johns Hopkins University School of Medicine, Baltimore, MD USA; 28 Northwestern University, Feinberg School of Medicine, Chicago, IL USA; 29 Autoimmune Diseases Research Unit, Department of Internal Medicine, BioCruces Health Research Institute. Hospital Universitario Cruces, University of the Basque Country, Barakaldo, Bizkaia Spain; 30 Mount Sinai Hospital and University Health Network, University of Toronto, Toronto, ON Canada; 31 Department of Rheumatology, Landspitali University Hospital, Reykjavik, Iceland; 32 Rheumatology Unit, Department of Medicine, Karolinska University Hospital, Solna, Sweden; 33 Cedars-Sinai Medical Center/David Geffen School of Medicine at UCLA, Los Angeles, CA USA; 34 Department of Rheumatology Hairmyres Hospital, East Kilbride, Scotland, UK

**Keywords:** Systemic lupus erythematosus, Neuropsychiatric, Nucleosomes, Nitration

## Abstract

**Background:**

In patients with systemic lupus erythematosus (SLE) there is no serological test that will reliably distinguish neuropsychiatric (NP) events due to active SLE from those due to other causes. Previously we showed that serum levels of nitrated nucleosomes (NN) were elevated in a small number of patients with NPSLE. Here we measured serum NN in samples from a larger population of patients with SLE and NP events to see whether elevated serum NN could be a marker for NPSLE.

**Methods:**

We obtained serum samples from patients in the Systemic Lupus International Collaborative Clinics (SLICC) inception cohort. This included 216 patients with NP events and two matched controls with SLE but no NP events for each of these patients. For the NP patients we tested samples taken before, during and after the NP event.

**Results:**

Twenty-six patients had events attributed to SLE according to the most stringent SLICC attribution rule. In these patients there was no association between onset of event and elevated serum NN. In 190 patients in whom events were not attributed to SLE by the SLICC rules, median serum NN was elevated at the onset of event (*P* = 0.006). The predominant clinical features in this group of 190 patients were headache, mood disorders and anxiety.

**Conclusions:**

Serum NN levels rise at the time of an NP event in a proportion of patients with SLE. Further studies are needed to determine the value of serum NN as a biomarker for NPSLE.

## Background

Neuropsychiatric manifestations of systemic lupus erythematosus (NPSLE) can take many different forms as defined by the American College of Rheumatology (ACR) case definitions in 1999 [1]. A study from the Systemic Lupus International Collaborative Clinics (SLICC) inception cohort in 2011 showed that 40% of 1206 newly diagnosed patients with SLE followed for a mean of 1.9 years developed at least one NP event [2]. Those with NP events had reduced quality of life, but the majority of these events were not attributed to SLE.

The NP events in the SLICC study were defined as either attributable to SLE or not attributable to SLE according to pre-determined classification rules [2]. For example, all isolated headaches (not occurring in association with another NPSLE event) were classified as not being attributable to SLE. Only a minority (30%) of the 843 NP events analysed were attributed to SLE, but these events were more likely to resolve than those not due to SLE [2]. This observation underlines the importance of developing biomarkers to identify those NP events that are caused by SLE. Currently there are no serological or imaging markers that will do this accurately, though an association between antiphospholipid antibodies and cerebrovascular events has been reported [3]. Potential biomarkers for NPSLE that have previously been studied are autoantibodies, cytokines or chemokines (reviewed in [4]). Despite a large number of studies over the last 30 years none of these biomarkers has shown a robust association with either NPSLE in general or with any specific NPSLE manifestation [4]. Similarly, a multi-centre report of neuroimaging appearances in 112 patients with NPSLE [5] identified no specific findings on magnetic resonance imaging (MRI) that clearly characterise NPSLE. The most common appearance was of non-specific vascular abnormalities.

In a recent paper we used a novel capture enzyme-linked immunosorbent assay (ELISA) to measure levels of nitrated nucleosomes (NN) in patients with SLE. Nucleosomes are released from apoptotic cells and the removal of this apoptotic debris is retarded in patients with SLE [6]. Both nucleosome level and anti-nucleosome antibodies are elevated in patients with SLE [7] and the latter are associated with disease activity, especially lupus nephritis [8]. Williams et al. used a monoclonal murine anti-nucleosome antibody 4H7 to assay serum nucleosome levels in 140 patients with SLE. They showed that nucleosome levels are strongly associated with disease activity measured by the Systemic Lupus Erythematosus Disease Activity Index (SLEDAI) score and were particularly high in patients with active nephritis or NPSLE [9]. Deposits of extracellular chromatin are found in renal biopsies from patients with lupus nephritis [10].

Nitric oxide (NO) produced by the endothelium [11] can cause irreversible nitration of tyrosine residues within proteins. Levels of serum NO are elevated in patients with SLE [11] and nitrotyrosine levels are higher in patients with active lupus nephritis than in those without nephritis [12].

Thus, since both nitration and nucleosome levels are associated with active SLE, our hypothesis was that levels of NN could be a marker of disease activity in patients with SLE. We measured serum NN in 397 samples taken longitudinally from 49 patients (mean 8 samples per patient) with SLE at times of different disease activity [13]. Serum NN levels were significantly higher in patients with SLE than in healthy control subjects or patients with other autoimmune rheumatic diseases [13]. However, only a subgroup (n = 31) of the patients with SLE had detectable serum NN at any time point. In those 31 patients, serum NN did vary over time but not in parallel with disease activity. Patients with NPSLE flares (defined as scoring A or B in the neuropsychiatric domain of the British Isles Lupus Assessment Group (BILAG) index) had mean NN twice the level in those without NP flares. However, this difference was based on only 18 samples from 11 patients and was not statistically significant [13].

To extend this research, we obtained serum samples from patients in the large SLICC inception cohort with documented NP events, and control samples from patients in the cohort without NP events. We tested serum NN in all patients, including longitudinal samples taken before, during and after the NP event. The objective was to determine if a transient rise in serum NN occurred concurrently with an NP event.

## Methods

All samples were obtained from patients enrolled in the SLICC inception cohort. This cohort has been described in previous papers [2, 3, 14]. Briefly, it comprises over 1600 patients recruited within 15 months of being diagnosed with SLE, from 31 centres across North America, Europe and Asia. All patients gave informed consent and ethical approval for the study was obtained by the institutional research ethics review board at each participating centre. Clinical data and blood samples from these patients were collected annually and submitted to the study centres in Toronto and Halifax, Canada.

### Neuropsychiatric (NP) events

An enrolment window extended from 6 months prior to the diagnosis of SLE up to the actual enrolment date. NP events were characterized within this window using the ACR case definitions for 19 NP syndromes [1]. These events were diagnosed by clinical evaluation supported by investigations, if clinically warranted, as per the ACR guidelines. Patients were reviewed annually within a 6-month window around the anniversary of enrolment. New NP events (since the last study visit) and the status of previous NP events since the last study visit were determined at each assessment.

### Attribution of NP events

In keeping with other publications on NP events within the SLICC NPSLE inception cohort, the same decision rules were used to determine the attribution of all NP events [2, 15]. To optimize consistency this determination was performed at the central coordinating centre in Halifax using data provided in the case record form by individual SLICC sites. Factors considered in the decision rules included: (i) the time of onset of NP event(s) in relation to the diagnosis of SLE; (ii) concurrent non-SLE factor(s), identified from the ACR glossary, which accompanied the case definitions of NP events [1] as potential causes (“exclusions”) or contributing factors (“associations”) for each NP syndrome and (iii) “common” NP events that are frequent in normal population controls as described by Ainiala et al. [16]. These included all isolated headaches, mild depression (mood disorders failing to meet criteria for “major depressive-like episodes”), anxiety, mild cognitive impairment (deficits in fewer than three of the eight specified cognitive domains) and polyneuropathy without electrophysiological confirmation. Two attribution decision rules of different stringency (models A and B) were developed as described in detail elsewhere [2, 15]. NP events that fulfilled criteria for model A (most stringent) or for model B (least stringent) were attributed to SLE. By definition, all NP events attributed to SLE using model A were included in the group of NP events using model B. Those events that did not fulfil these criteria were attributed to non-SLE causes (non-A, non-B events). Non-A, non-B events comprised 70% of all events [2].

We tested samples from 26 patients with model-A NP events and 190 with non-A, non-B events (i.e. 216 patients with NP events in total). For each of these patients we attempted to obtain and test three samples; one from the date closest to the event, one from before that date and one from after. These were defined as the onset, pre-event and post-event samples, respectively. For each of the 216 patients with NP events we tested samples from two age-matched and sex-matched controls from the inception cohort who had no recorded NP events. Only one sample from each control subject was tested. We also tested samples from 16 patients with a history of chronic migraine who did not have SLE or any other autoimmune rheumatic disease. These patients were recruited with informed consent from the Headache Clinic at the National Hospital for Neurology and Neurosurgery, Queen Square, London, UK. In our previous paper [13] we showed that none of 37 healthy controls had any detectable serum NN, so we did not repeat testing of healthy controls in this study.

#### Assay for measuring nitrated nucleosomes

The whole assay was done at room temperature (RT) except where specified and plates were washed three to four times with PBS-0.1% Tween (PBST) between steps: 96-well streptavidin plates were divided in half and the test side was coated with biotinylated polyclonal goat anti-nitrotyrosine antibody (Abcam 27646) diluted 1:1000 in PBS, and the control side coated with PBS (75 μL per well). After one hour incubation plates were washed and blocked with 200 μL of 0.5% ovalbumin in PBST (OVA-BST) for one hour. After washing, serum samples were loaded in duplicate onto the plates (100 μL/well) at 1:30 dilution in PBS, such that each sample was loaded in two wells on the test side and two matching wells on the control side. After one hour incubation at 37 °C and washing, 50 μL per well of rabbit anti-histone H3 antibody (sc-10809, Santa Cruz Biotechnology) diluted at 1:2000 in OVA-BST was added and the plates were incubated for one hour. After washing, 50 μL per well of goat anti-rabbit IgG horse radish peroxidase (HRP) conjugate (Dako P0448) diluted at 1:2000 in 0.5% OVA-BST was added. After incubating for one hour and washing, HRP substrate was added (100 μL per well) and incubated for 10 min. The reaction was stopped with 100 μL sulphuric acid and optical density (OD) read at 450 nm. The net optical density (OD) reading for each sample was calculated by subtracting the OD in the control well from that in the matching test well to exclude non-specific background binding.

In order to be able to compare OD values obtained from different plates on different days we prepared an in-house standard positive control sample that was loaded in serial dilutions (range 1:15–1:120) on every plate. This in-house standard was prepared by pooling serum samples from several patients who had been found to have high serum NN levels in this assay. The mean net OD from duplicate test samples was converted to absorbance units (AU) by comparison to the standard curve of OD for the serial dilutions of the positive control sample on each plate; 100 AU was defined as the OD given by a 1:30 dilution of the positive control sample. The OD for this sample was reproducibly high, ranging between 1.03 and 1.37. This assay was reproducible with an intra-plate and inter-plate coefficient of variation of < 10%.

#### Statistical analysis

All statistical analysis was done using Prism Graphpad 6.0® (GraphPad Software Inc, La Jolla, CA 92037 USA). The main outcome measure was serum NN, which did not follow a normal distribution (Shapiro-Wilk normality test). The highly positive skewed distribution could not be transformed to a normally distributed scale, thus the outcomes were assumed to follow a negative binomial distribution and non-parametric tests were selected. Variables measured at the patient level were compared between groups using either Fisher’s exact test for the categorical variables or the unpaired *t* test for continuous variables. Continuous variables with a positively skewed distribution were log-transformed before the analysis.

## Results

Table 1 shows the demographic and medication data for each of the subject groups. Table 2 shows the type of NP event in the model A and the non-A, non-B NP event groups. As would be expected from the SLICC attribution rules, the groups are very different. In the non-A, non-B group 185/190 events were headache, anxiety disorder or mood disorder, whereas these three manifestations accounted for only one case in the model-A group.

**Table 1:** Demographic and treatment characteristics of patients with SLE studied at the time of the onset sample

	Model-A NP event	Matched controls for model-A NP events	Non-A, non-B NP events	Matched controls for non-A, non-B NP events
Number of subjects	26	52	190	380
Mean (SD) age	34.4 (7.5)	36.9 (10.9)	33.3 (11.8)	35.3 (11.5)
Gender (female:male)	21:5	42:10	177:13	353:27
Ethnicity	9 W, 6 A-C, 4 SA, 4 EA, 3 H	22 W, 12 A-C, 4 SA, 7 EA, 6 H, 1 other	84 W, 30 A-C, 4 SA, 41 EA, 18 H, 13 other	161 W, 61 A-C, 13 SA, 81 EA, 36 H, 26 other, 2 unknown
Median dose of corticosteroids (mg)	10	0.25	7.5	5
Number (%) taking anti-malarials	16 (61)	22 (42)	140 (74)	222 (58)
Number (%) taking immunosuppressant drugs	13 (50)	10 (19)	71 (37)	144 (38)

Demographic and clinical characteristics of the patients with systemic lupus erythematosus (SLE) studied in all four groups. *NP* neuropsychiatric, *W* white, *A-C* Afro-Caribbean, *EA* East Asian, *SA* South Asian, *H* Hispanic

**Table 2:** Type of NP event in patients with model A compared to non-A, non-B events

Type of NP event	Number of events in model-A group (total n = 26)	Number of events in non-A, non-B group (total n = 190)
Acute inflammatory demyelinating polyneuropathy	1	0
Aseptic meningitis	1	1
Cerebrovascular disease	2	0
Headache	0	157
Mononeuropathy	3	1
Cranial neuropathy	4	0
Polyneuropathy	1	0
Seizures	7	2
Acute confusional state	1	1
Anxiety disorder	0	10
Cognitive dysfunction	1	0
Mood disorders	1	18
Psychosis	4	0

The table shows the nature of the neuropsychiatric (NP) events comparing patients in the model-A and non-A, non-B groups

Considering all 216 patients who had events, the median time between the date of the NP event and the date of the onset sample was 2 months (maximum 6 months). Of the onset samples, 67 came from a time point before the event and 149 from after the event.

### Patients with model-A NP events

**Fig. 1:**
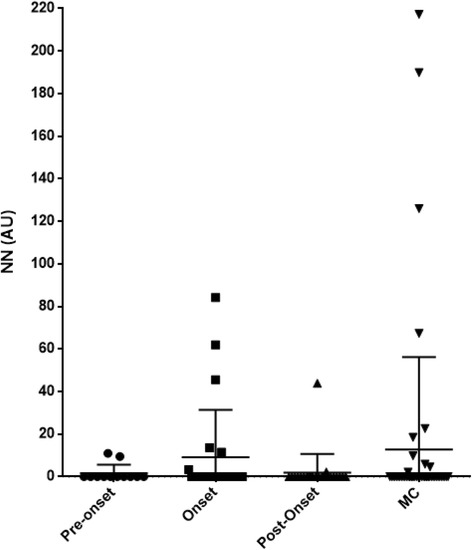
Serum nitrated nucleosomes (NN) levels in pre-event, onset and post-model-A event samples compared to matched controls (MC). Pre-event samples were available for 12 patients, onset samples for 24 patients and post-event samples for 25 patients. *AU* absorbance units

Figure 1 shows that there were no significant differences between median serum NN in the pre-event, onset and post-event samples or matched controls (*P* = 0.46, one-way analysis of variance (ANOVA)).

### Patients with non-A, non-B NP events

**Fig. 2:**
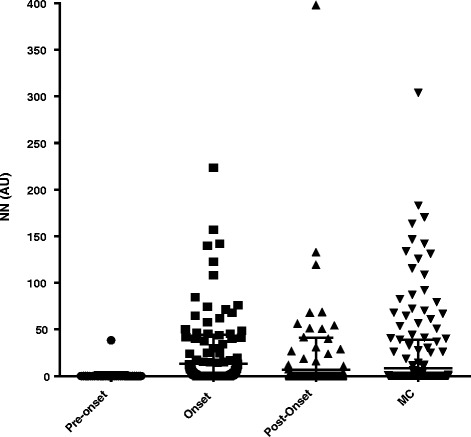
Serum nitrated nucleosomes (NN) levels in pre-event, onset and post-non-A, non-B event samples compared to matched controls (MC). Pre-event samples were available for 106 patients, onset samples for 182 patients and post-event samples for 181 patients. *AU* absorbance units

Figure 2 shows that median serum NN was highest in the onset sample compared to pre-event or post-event samples (*P* = 0.006, one-way ANOVA). Mean serum NN for onset samples was also significantly higher than in the matched controls (*P* < 0.001, Wilcoxon test).

**Fig. 3:**
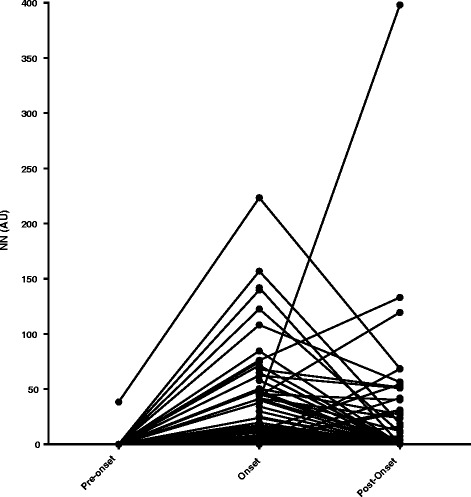
Changes in serum nitrated nucleosomes (NN) levels of patients with non-A, non-B neuropsychiatric events over time. *AU* absorbance units

Figure 3 shows the pattern of serum NN levels over time in all 190 patients with non-A, non-B NP events, illustrating the rise at the time of the onset sample, followed by a fall, in some patients.

Since headache was the dominant clinical feature in the non-A, non-B group (157/190 patients (83%)), we investigated whether rise in serum NN is a non-specific effect associated with headache in general. We tested samples from 16 patients with chronic migraine who had no known autoimmune rheumatic disease (mean age 48.3 years, 15 white, 9/16 female). None of these patients had any detectable serum NN. In our previous paper we found no detectable serum NN in 37 healthy controls and 11/13 patients with rheumatoid arthritis, 11/12 with myositis and 9/13 with Sjogrens’ syndrome, so overall it seems unlikely that a rise in serum NN occurs commonly as a non-specific event outside the context of SLE [13].

We also tested for any association between serum NN and the following measures of disease activity; C3, anti-dsDNA, global SLEDAI score, SLEDAI score excluding serology (anti-DNA and complement), renal SLEDAI and SLEDAI excluding NP variables. There was no association between serum NN and any of these variables in either group of patients with NP events (model A or non-A, non-B) or the matched controls. When the analysis was restricted only to patients with headaches or only to patients with serum NN above the 90^th^ percentile of healthy controls there were still no associations.

## Discussion

Apart from the recognised association between antiphospholipid antibodies and cerebrovascular disease [3], biomarkers previously thought to show great promise in the management of NPSLE, such as anti-ribosomal P antibodies [17] and anti N-methyl-D-aspartate receptor antibodies [18], have not come into widespread clinical use. It is difficult to validate any putative biomarker in NPSLE because it is a rare condition and because many patients with SLE have NP events not caused by the disease itself [2]. Our previous study had raised the possibility that elevated serum NN could be a marker of NPSLE, but was limited by small numbers of patients and a limited variety of NP events. In order to address these limitations, we accessed samples and clinical data from the SLICC inception cohort, which is one of the largest populations in which comprehensive data on NPSLE events are available. Even in this multi-national cohort of over 1600 patients, we were only able to study 26 patients with model-A NP events and for half of these 26 patients we did not have a full set of pre-event, onset and post-event samples. We could not show any relationship between elevated serum NN and NP events in these 26 patients.

Our original purpose in including patients with NP events not attributed to SLE was as a control group in which we did not expect to see a rising and falling pattern of serum NN. However, in the event, the serum NN level was statistically higher at event onset in this group than in the pre-event or post-event samples or in the matched controls. We did not detect any similar statistically significant result in patients with model-A NP events, perhaps due to the much smaller numbers in that group.

We considered a number of possible explanations for these serum NN results in patients with non-A, non-B events. The highly significant *P* values suggest that this was not a random or chance finding. These were not simply individuals with constitutively high levels of serum nitration, as the pre-event samples had very low serum NN levels. Since the majority of the events were headaches it was possible that elevated serum NN is a non-specific effect of headache, but this seems unlikely because we found no NN in serum from any patients who suffer chronic headache but do not have lupus. It was also possible that the rise in serum NN could have been due to lupus activity in non-NP systems but we found no association between serum NN and any marker of global, serological or organ-specific disease activity that was assessed in this paper (in any of the patient groups). Nor did we find such associations in our previous paper [13].

Under conditions of systemic inflammation, excess nitric oxide (NO) produced by the vascular endothelium [11] can cause irreversible nitration of tyrosine residues on serum proteins including histones in nucleosomes. NO is also believed to contribute to the causation of headaches through its effects on cerebral and extra-cerebral cranial blood flow [19]. In patients with SLE, serum nitrite plus nitrate level (an index for NO production) correlates with disease activity and levels of anti-dsDNA antibodies [11, 12]. There are also potential links between nitration and NPSLE. Human astrocytes are known to express inducible nitric oxide synthase (iNOS) which can be stimulated by cytokines including interleukin-1β and interferon gamma [20]. A Swedish group showed in 1998 that total cerebrospinal fluid (CSF) (nitrite + nitrate) was higher in patients with NPSLE than in age-matched and sex-matched controls and higher in patients with severe compared to those with mild NPSLE [21]. In a subsequent study of 30 patients who had NPSLE (with clinical features fulfilling the ACR case definitions [1]), the same group confirmed significantly higher CSF (nitrate + nitrite) in 7 patients with severe NPSLE (psychosis, dementia, combinations of several NP manifestations) compared to 23 patients with mild or moderate NPSLE (including cognitive impairment, seizures, headache, anxiety, mood disorders) [22].

The concept of lupus headache is complex and many papers have suggested that it is of limited usefulness because headache is such a common complaint (in people with or without SLE). For example, in a meta-analysis, Mitsikostas et al. showed that although headache occurs in 50% of patients with SLE, this is not significantly different from the population prevalence of this very common symptom [23]. Furthermore there was no clear evidence that headache in patients with SLE was related to disease activity or arose from a specific autoimmune or inflammatory mechanism [23]. Analysis of headaches in the SLICC inception cohort showed a prevalence of 17.8% at enrolment and that 58% of subjects suffered headache at some time over a 10-year follow-up period [14]. Only 27 of 697 headaches were identified as lupus headaches by the treating physician. However, there was no consensus on the characteristics of headache in this group as reflected by the headache subtypes in the case definitions. Of these 27 lupus headaches, 26 resolved over a mean follow-up period of 3.8 years [14].

There are a number of limitations affecting our analysis. As noted previously, we did not have a full complement of samples for most of the patients with model-A events. Since clinical data and blood samples are obtained annually for the SLICC study, the time period between the pre-event, onset and post-event samples ranged from a few months to several years. However, the onset samples were from time points close to the event (median gap 2 months, maximum 6 months). In our previous smaller study in 49 patients, higher serum NN was seen in patients on hydroxychloroquine or immunosuppressant drugs. In the current study, the patients with model-A NP events were on higher corticosteroid doses and were more likely to be taking immunosuppressant drugs than the matched controls (Table 1), but there were no corresponding differences between the patients with non-A, non-B events and their controls. We considered obtaining further samples from patients who had NP events that were due to SLE under model B but not model A. However, the study centre in Toronto informed us that there were only eight such subjects for whom sufficient samples were available and this was not pursued.

Although we have used the term nitrated nucleosomes in this paper, we stressed in our previous paper and repeat here that our assay could potentially detect any analyte that contains both histones and nitrotyrosine, for example nucleosome fragments or nitrated free histones. However, published evidence shows that circulating free histones are rarely found except in conditions such as severe trauma [24]. Specifically, although the presence of circulating nucleosomes in blood has been established for many years [7, 25], sub-nucleosome components are rapidly cleared through the liver and thus are rarely detectable [26]. In fact circulating histones are toxic to endothelial cells [24]. It is important to be clear, however, that this assay has not been validated using purified nucleosomes and this validation would be an important step before it could be used in clinical practice.

Use of the serum NN assay clinically cannot yet be advocated based on these results alone. Though the capture ELISA is reproducible in our hands, with inter-plate and intra-plate variation < 10%, it has not yet been carried out elsewhere and the standard positive control used to quantify the results is a blend of serum from our patients..

## Conclusions

In previous studies both nitration and nucleosomes have been found to be elevated in association with active SLE. In this multi-centre study we found that serum NN levels rise at the time of an NP event in a proportion of patients with SLE, including some cases of headache. Further studies are needed to determine the value of serum NN as a biomarker for NPSLE. The NN assay will require further validation and standardisation of the nature and biochemistry of the antigen being recognised.

## Abbreviations

ACR: American College of Rheumatology
ANOVA: Analysis of variance
AU: Absorbance units
BILAG: British Isles Lupus Assessment Group
ELISA: Enzyme-linked immunosorbent assay
HRP: Horseradish peroxidase
iNOS: Inducible nitric oxide synthase
NN: Nitrated nucleosomes
NO: Nitric oxide
NP: Neuropsychiatric
OD: Optical density
PBS: Phosphate-buffered saline
SLE: Systemic lupus erythematosus
SLEDAI: Systemic lupus erythematosus disease activity index
SLICC: Systemic Lupus International Collaborative Clinics

## References

[1] The American College of Rheumatology nomenclature and case definitions for neuropsychiatric lupus syndromes. Arthritis Rheum. 1999;42(4):599–608. 10.1002/1529-0131(199904)42:4<599::AID-ANR2>3.0.CO;2-F 10211873

[2] Hanly JG, Urowitz MB, Su L, Bae SC, Gordon C, Wallace DJ, Clarke A, Bernatsky S, Isenberg D, Rahman A (2010). Prospective analysis of neuropsychiatric events in an international disease inception cohort of patients with systemic lupus erythematosus. Ann Rheum Dis.

[3] Hanly JG, Urowitz MB, Su L, Bae SC, Gordon C, Clarke A, Bernatsky S, Vasudevan A, Isenberg D, Rahman A (2011). Autoantibodies as biomarkers for the prediction of neuropsychiatric events in systemic lupus erythematosus. Ann Rheum Dis.

[4] Jeltsch-David H, Muller S (2014). Neuropsychiatric systemic lupus erythematosus: pathogenesis and biomarkers. Nat Rev Neurol.

[5] Sarbu N, Alobeidi F, Toledano P, Espinosa G, Giles I, Rahman A, Yousry T, Capurro S, Jager R, Cervera R (2015). Brain abnormalities in newly diagnosed neuropsychiatric lupus: systematic MRI approach and correlation with clinical and laboratory data in a large multicenter cohort. Autoimmun Rev.

[6] Munoz LE, Gaipl US, Franz S, Sheriff A, Voll RE, Kalden JR, Herrmann M (2005). SLE–a disease of clearance deficiency?. Rheumatology (Oxford).

[7] Amoura Z, Piette JC, Chabre H, Cacoub P, Papo T, Wechsler B, Bach JF, Koutouzov S (1997). Circulating plasma levels of nucleosomes in patients with systemic lupus erythematosus: correlation with serum antinucleosome antibody titers and absence of clear association with disease activity. Arthritis Rheum.

[8] Manson JJ, Ma A, Rogers P, Mason LJ, Berden JH, van der Vlag J, D'Cruz DP, Isenberg DA, Rahman A (2009). Relationship between anti-dsDNA, anti-nucleosome and anti-alpha-actinin antibodies and markers of renal disease in patients with lupus nephritis: a prospective longitudinal study. Arthritis Res Ther.

[9] Williams RC, Malone CC, Meyers C, Decker P, Muller S (2001). Detection of nucleosome particles in serum and plasma from patients with systemic lupus erythematosus using monoclonal antibody 4H7. J Rheumatol.

[10] Kalaaji M, Fenton KA, Mortensen ES, Olsen R, Sturfelt G, Alm P, Rekvig OP (2007). Glomerular apoptotic nucleosomes are central target structures for nephritogenic antibodies in human SLE nephritis. Kidney Int.

[11] Belmont HM, Levartovsky D, Goel A, Amin A, Giorno R, Rediske J, Skovron ML, Abramson SB (1997). Increased nitric oxide production accompanied by the up-regulation of inducible nitric oxide synthase in vascular endothelium from patients with systemic lupus erythematosus. Arthritis Rheum.

[12] Oates JC, Christensen EF, Reilly CM, Self SE, Gilkeson GS (1999). Prospective measure of serum 3-nitrotyrosine levels in systemic lupus erythematosus: correlation with disease activity. Proc Assoc Am Physicians.

[13] Croca S, Bassett P, Pericleous C, Alber KF, Latchman D, Isenberg D, Giles I, Rahman A, Ioannou Y (2014). Serum nitrated nucleosome levels in patients with systemic lupus erythematosus: a retrospective longitudinal cohort study. Arthritis Res Ther.

[14] Hanly JG, Urowitz MB, O'Keeffe AG, Gordon C, Bae SC, Sanchez-Guerrero J, Romero-Diaz J, Clarke AE, Bernatsky S, Wallace DJ (2013). Headache in systemic lupus erythematosus: results from a prospective, international inception cohort study. Arthritis Rheum.

[15] Hanly JG, Urowitz MB, Sanchez-Guerrero J, Bae SC, Gordon C, Wallace DJ, Isenberg D, Alarcon GS, Clarke A, Bernatsky S (2007). Neuropsychiatric events at the time of diagnosis of systemic lupus erythematosus: an international inception cohort study. Arthritis Rheum.

[16] Ainiala H, Hietaharju A, Loukkola J, Peltola J, Korpela M, Metsanoja R, Auvinen A (2001). Validity of the new American College of Rheumatology criteria for neuropsychiatric lupus syndromes: a population-based evaluation. Arthritis Rheum.

[17] Karassa FB, Afeltra A, Ambrozic A, Chang DM, De Keyser F, Doria A, Galeazzi M, Hirohata S, Hoffman IE, Inanc M (2006). Accuracy of anti-ribosomal P protein antibody testing for the diagnosis of neuropsychiatric systemic lupus erythematosus: an international meta-analysis. Arthritis Rheum.

[18] Lauvsnes MB, Omdal R (2012). Systemic lupus erythematosus, the brain, and anti-NR2 antibodies. J Neurol.

[19] Olesen J (2008). The role of nitric oxide (NO) in migraine, tension-type headache and cluster headache. Pharmacol Ther.

[20] Lee SC, Dickson DW, Liu W, Brosnan CF (1993). Induction of nitric oxide synthase activity in human astrocytes by interleukin-1 beta and interferon-gamma. J Neuroimmunol.

[21] Brundin L, Svenungsson E, Morcos E, Andersson M, Olsson T, Lundberg I, Wiklund NP (1998). Central nervous system nitric oxide formation in cerebral systemic lupus erythematosus. Ann Neurol.

[22] Svenungsson E, Andersson M, Brundin L, van Vollenhoven R, Khademi M, Tarkowski A, Greitz D, Dahlstrom M, Lundberg I, Klareskog L (2001). Increased levels of proinflammatory cytokines and nitric oxide metabolites in neuropsychiatric lupus erythematosus. Ann Rheum Dis.

[23] Mitsikostas DD, Sfikakis PP, Goadsby PJ (2004). A meta-analysis for headache in systemic lupus erythematosus: the evidence and the myth. Brain.

[24] Abrams ST, Zhang N, Manson J, Liu T, Dart C, Baluwa F, Wang SS, Brohi K, Kipar A, Yu W (2013). Circulating histones are mediators of trauma-associated lung injury. Am J Respir Crit Care Med.

[25] Rumore PM, Steinman CR (1990). Endogenous circulating DNA in systemic lupus erythematosus. Occurrence as multimeric complexes bound to histone. J Clin Invest.

[26] Gauthier VJ, Tyler LN, Mannik M (1996). Blood clearance kinetics and liver uptake of mononucleosomes in mice. J Immunol.

